# Supplementary material for: Urinary metabolite model to predict the dying process in lung cancer patients
Source: Commun Med (Lond). 2025 Feb 27;5:49. doi: 10.1038/s43856-025-00764-3 (PMC11868640; doi:10.1038/s43856-025-00764-3)
Supplement: Supplementary file 2 — Supplementary information [file 43856_2025_764_MOESM2_ESM.pdf]

Supplementary Information

Title:

Urinary metabolite model to predict the dying process in lung cancer patients

Supplementary Figures:

Supplementary Figure 1 - 10 Comparison of each metabolite identified by ANOVA over the last 12 weeks of life versus more than 3 months from death.

Supplementary Figure 11 Calibration curves for the 30 day Cox lasso regression model at days 10, 20 and 30 in the Training cohort and Validation cohort.

Supplementary Tables:

Supplementary Table 1 Table of molecular mass and Retention Time (RT) for the Unknown metabolites.

Supplementary Table 2 MS2 Spectra data for Unknown Metabolite 5

Supplementary Table 3 MS2 Spectra data for Unknown Metabolite 7

Supplementary Table 4 Metabolites identified for pathway analysis

Supplementary Table 5 KEGG Pathway analysis

Supplementary Figure 1

Comparison of each metabolite identified by ANOVA over the last 12 weeks of life versus more than 3 months from death.

Plots for each metabolite identified as significant by ANOVA (i.e. FDR adjusted p-value <0·05.). Metabolites were identified from different LC-QTOF-MS protocols (A = Amide column, D= DC18 column, '+' = Positive mode, '-' = Negative mode). Each metabolite is compared over the last 12 weeks of life versus more than 3 months from death. Week 01 (n=26), 02 (n=18) and 03 (n=12) on the x-axis indicates patients' measurement in the last week, 2 weeks and 3 weeks of life; Week 04+ (n=15) indicates measurements from week 4 to week 11; Week 12+ (n=41) indicates more than 3 months before death. Data were normalized by reference feature, log-transformed and auto scaled (centered around the mean and divided by the standard deviation of each variable).

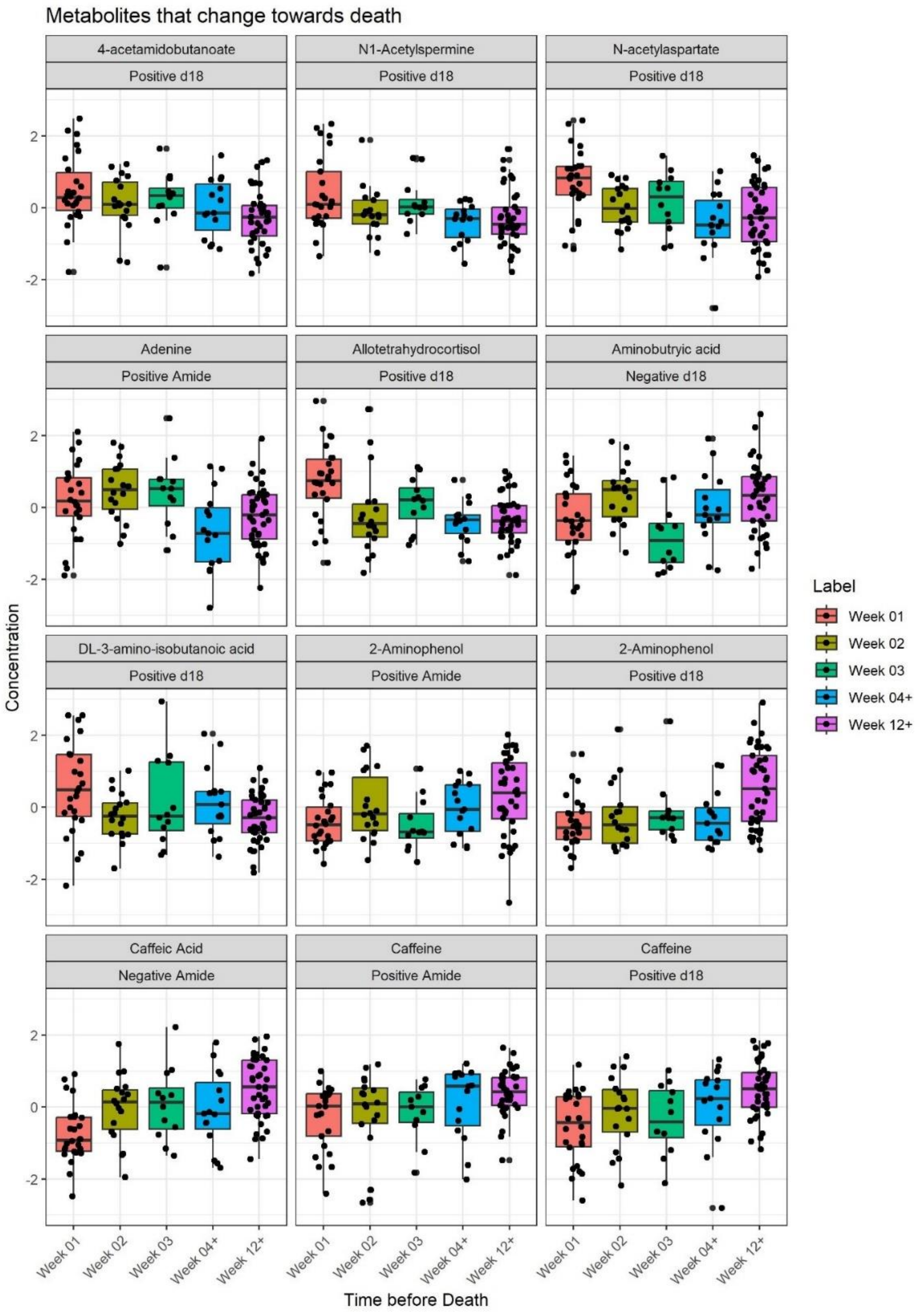

Supplementary Figure 2

Comparison of each metabolite identified by ANOVA over the last 12 weeks of life versus more than 3 months from death.

Plots for each metabolite identified as significant by ANOVA (i.e. FDR adjusted p-value <0·05.). Metabolites were identified from different LC-QTOF-MS protocols (A = Amide column, D= DC18 column, '+' = Positive mode, '-' = Negative mode). Each metabolite is compared over the last 12 weeks of life versus more than 3 months from death. Week 01 (n=26), 02 (n=18) and 03 (n=12) on the x-axis indicates patients' measurement in the last week, 2 weeks and 3 weeks of life; Week 04+ (n=15) indicates measurements from week 4 to week 11; Week 12+ (n=41) indicates more than 3 months before death. Data were normalized by reference feature, log-transformed and auto scaled (centered around the mean and divided by the standard deviation of each variable).

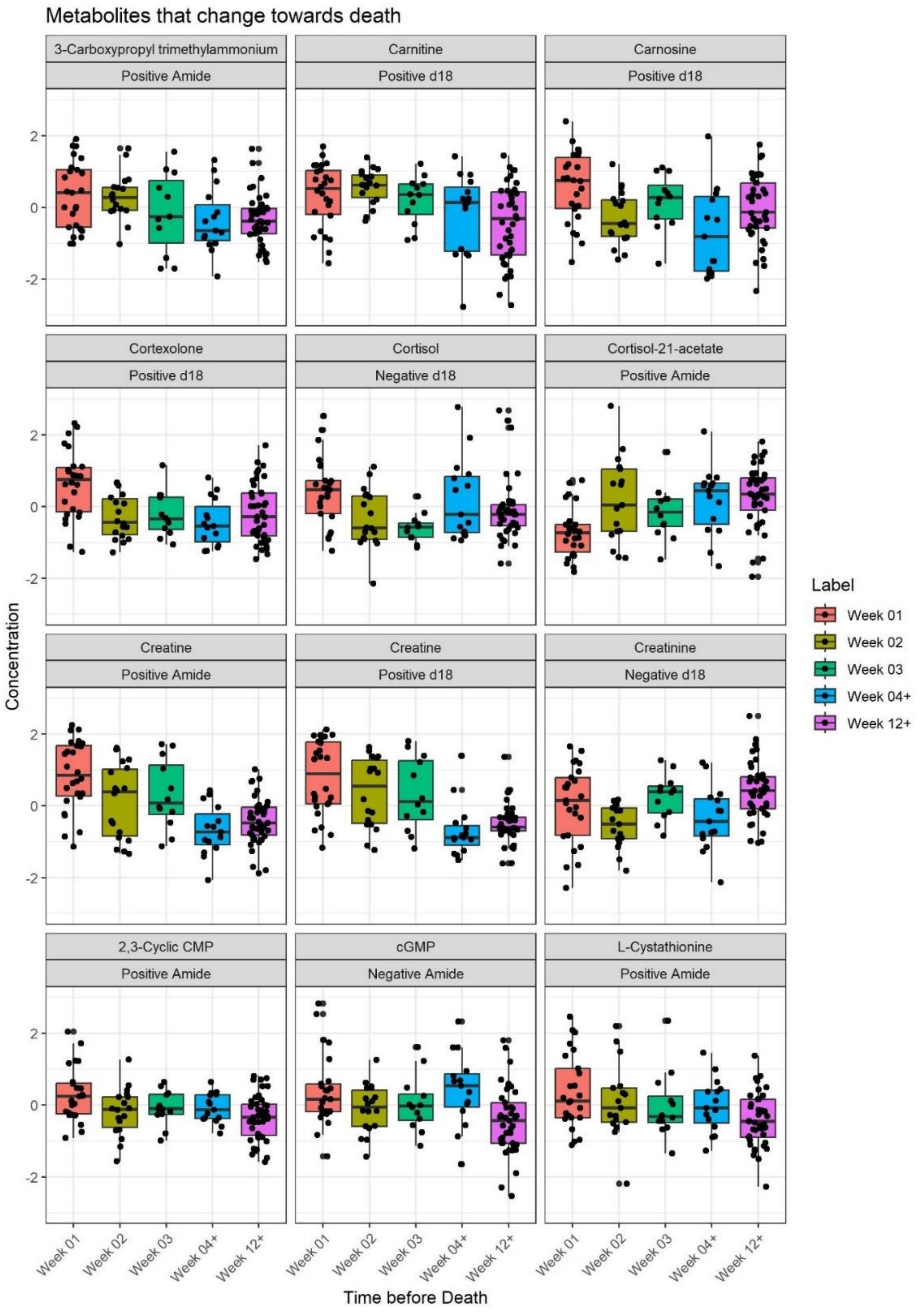

Supplementary Figure 3

Comparison of each metabolite identified by ANOVA over the last 12 weeks of life versus more than 3 months from death.

Plots for each metabolite identified as significant by ANOVA (i.e. FDR adjusted p-value <0·05.). Metabolites were identified from different LC-QTOF-MS protocols (A = Amide column, D= DC18 column, '+' = Positive mode, '-' = Negative mode). Each metabolite is compared over the last 12 weeks of life versus more than 3 months from death. Week 01 (n=26), 02 (n=18) and 03 (n=12) on the x-axis indicates patients' measurement in the last week, 2 weeks and 3 weeks of life; Week 04+ (n=15) indicates measurements from week 4 to week 11; Week 12+ (n=41) indicates more than 3 months before death. Data were normalized by reference feature, log-transformed and auto scaled (centered around the mean and divided by the standard deviation of each variable).

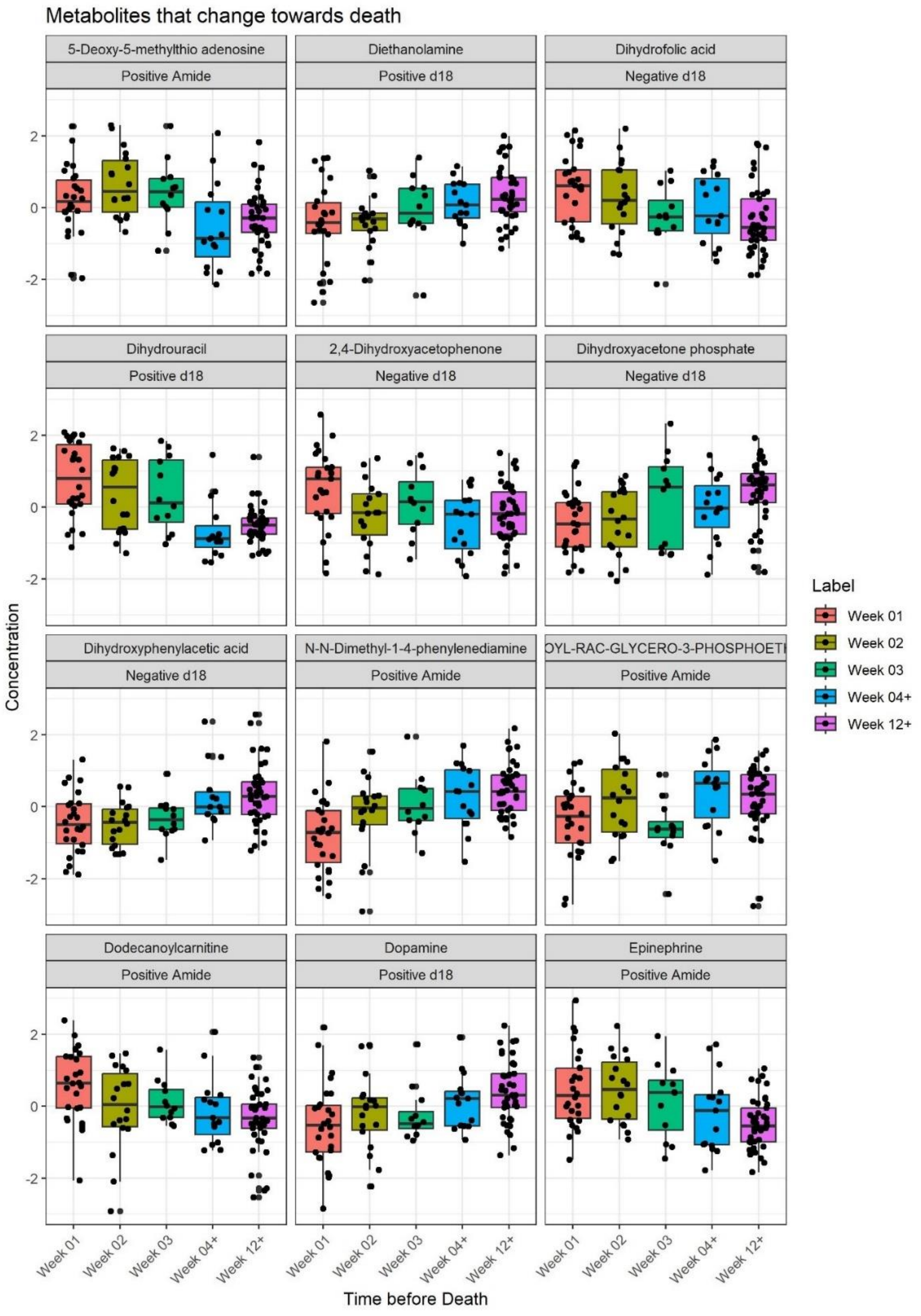

Supplementary Figure 4

Comparison of each metabolite identified by ANOVA over the last 12 weeks of life versus more than 3 months from death.

Plots for each metabolite identified as significant by ANOVA (i.e. FDR adjusted p-value <0·05.). Metabolites were identified from different LC-QTOF-MS protocols (A = Amide column, D= DC18 column, '+' = Positive mode, '-' = Negative mode). Each metabolite is compared over the last 12 weeks of life versus more than 3 months from death. Week 01 (n=26), 02 (n=18) and 03 (n=12) on the x-axis indicates patients' measurement in the last week, 2 weeks and 3 weeks of life; Week 04+ (n=15) indicates measurements from week 4 to week 11; Week 12+ (n=41) indicates more than 3 months before death. Data were normalized by reference feature, log-transformed and auto scaled (centered around the mean and divided by the standard deviation of each variable).

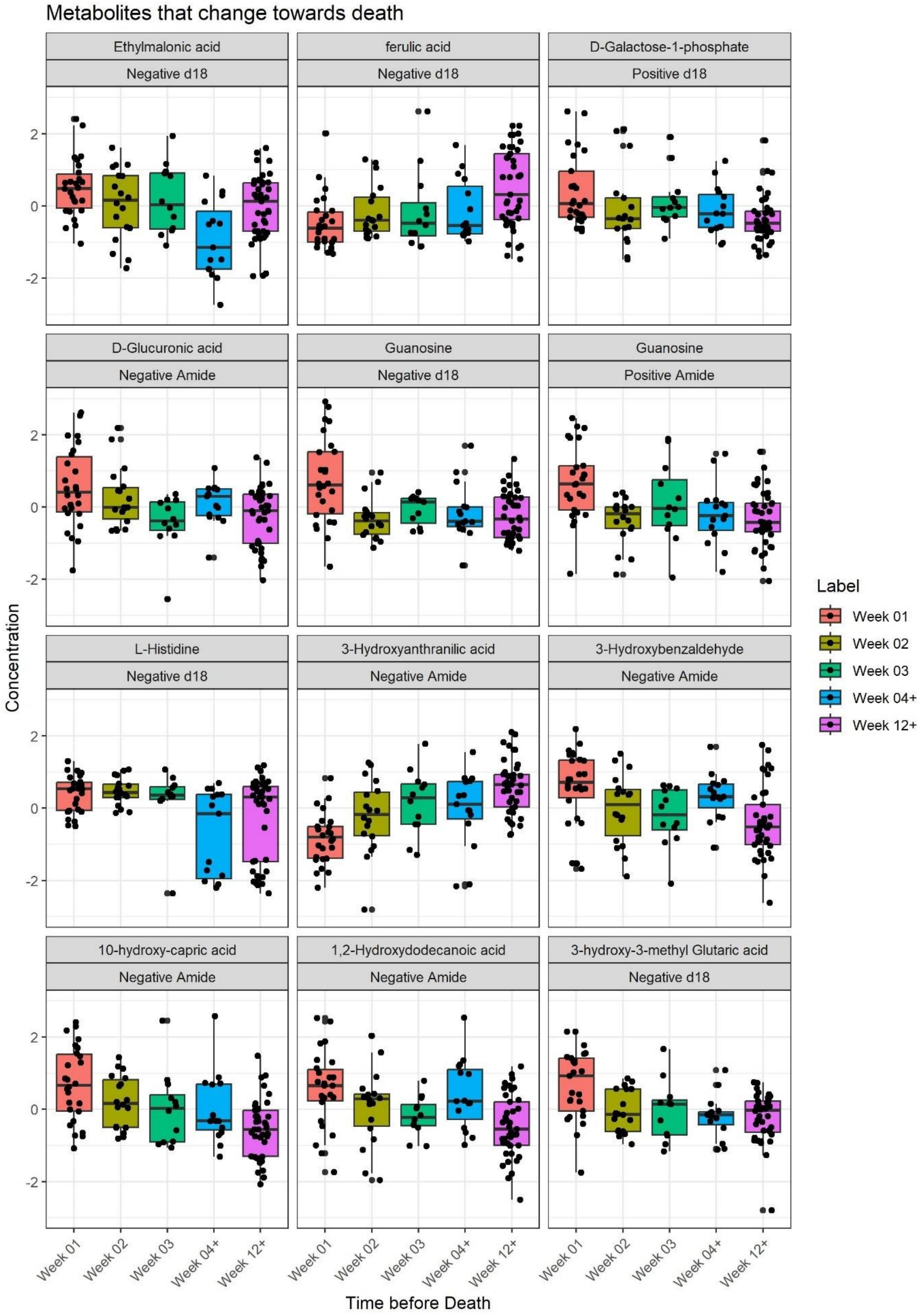

Supplementary Figure 5

Comparison of each metabolite identified by ANOVA over the last 12 weeks of life versus more than 3 months from death.

Plots for each metabolite identified as significant by ANOVA (i.e. FDR adjusted p-value <0·05.). Metabolites were identified from different LC-QTOF-MS protocols (A = Amide column, D= DC18 column, '+' = Positive mode, '-' = Negative mode). Each metabolite is compared over the last 12 weeks of life versus more than 3 months from death. Week 01 (n=26), 02 (n=18) and 03 (n=12) on the x-axis indicates patients' measurement in the last week, 2 weeks and 3 weeks of life; Week 04+ (n=15) indicates measurements from week 4 to week 11; Week 12+ (n=41) indicates more than 3 months before death. Data were normalized by reference feature, log-transformed and auto scaled (centered around the mean and divided by the standard deviation of each variable).

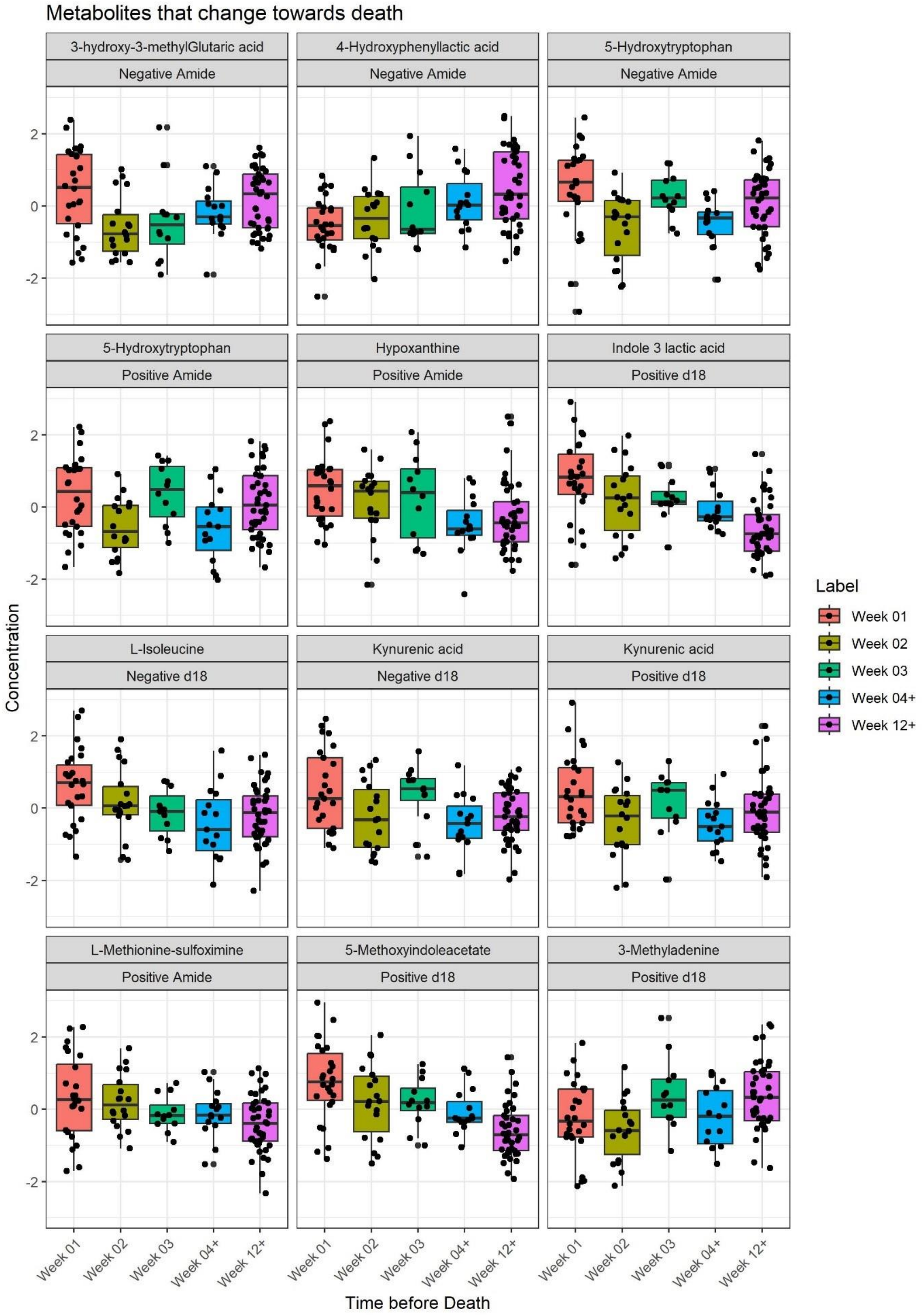

Supplementary Figure 6

Comparison of each metabolite identified by ANOVA over the last 12 weeks of life versus more than 3 months from death.

Plots for each metabolite identified as significant by ANOVA (i.e. FDR adjusted p-value <0·05.). Metabolites were identified from different LC-QTOF-MS protocols (A = Amide column, D= DC18 column, '+' = Positive mode, '-' = Negative mode). Each metabolite is compared over the last 12 weeks of life versus more than 3 months from death. Week 01 (n=26), 02 (n=18) and 03 (n=12) on the x-axis indicates patients' measurement in the last week, 2 weeks and 3 weeks of life; Week 04+ (n=15) indicates measurements from week 4 to week 11; Week 12+ (n=41) indicates more than 3 months before death. Data were normalized by reference feature, log-transformed and auto scaled (centered around the mean and divided by the standard deviation of each variable).

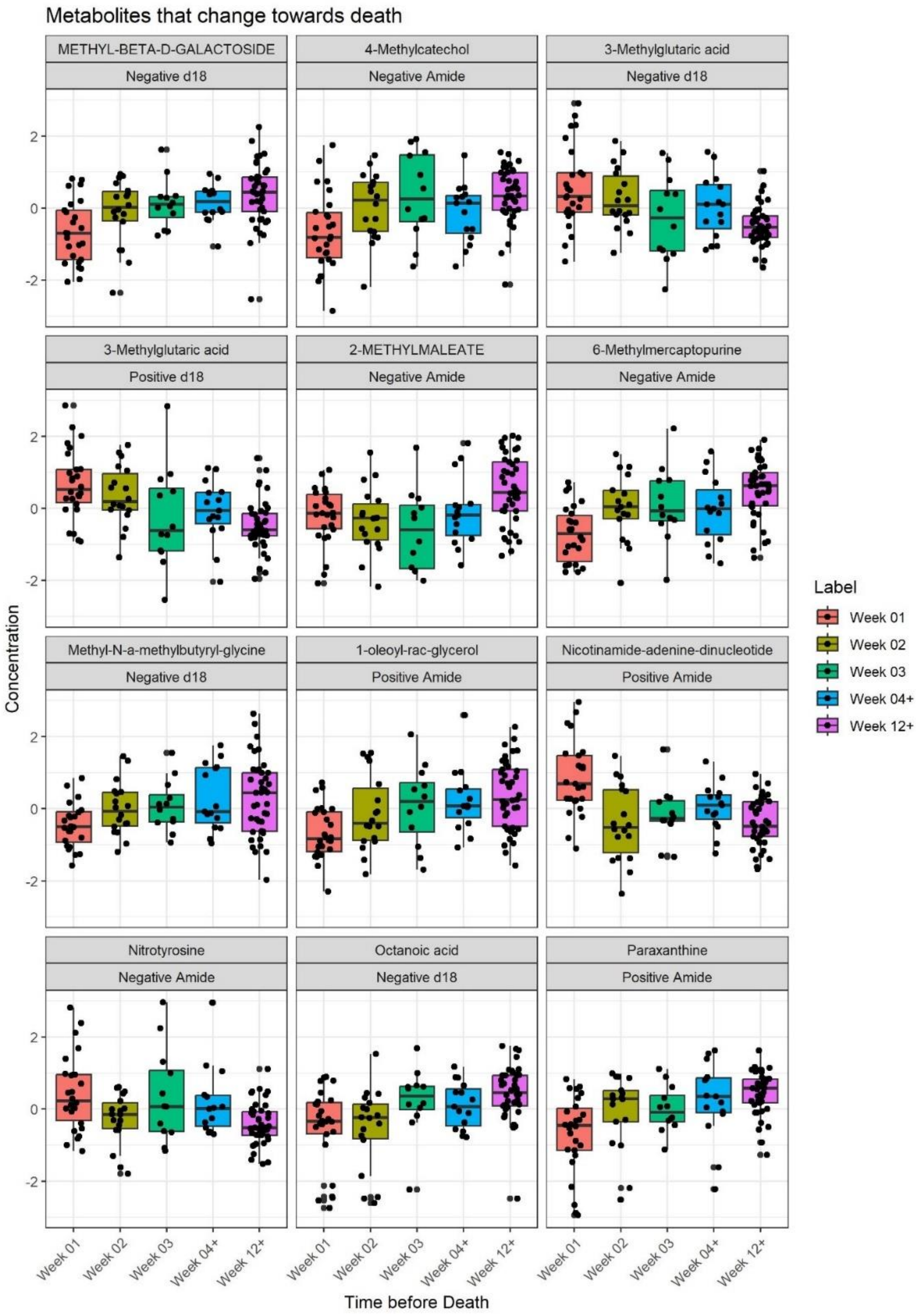

Supplementary Figure 7

Comparison of each metabolite identified by ANOVA over the last 12 weeks of life versus more than 3 months from death.

Plots for each metabolite identified as significant by ANOVA (i.e. FDR adjusted p-value <0·05.). Metabolites were identified from different LC-QTOF-MS protocols (A = Amide column, D= DC18 column, '+' = Positive mode, '-' = Negative mode). Each metabolite is compared over the last 12 weeks of life versus more than 3 months from death. Week 01 (n=26), 02 (n=18) and 03 (n=12) on the x-axis indicates patients' measurement in the last week, 2 weeks and 3 weeks of life; Week 04+ (n=15) indicates measurements from week 4 to week 11; Week 12+ (n=41) indicates more than 3 months before death. Data were normalized by reference feature, log-transformed and auto scaled (centered around the mean and divided by the standard deviation of each variable).

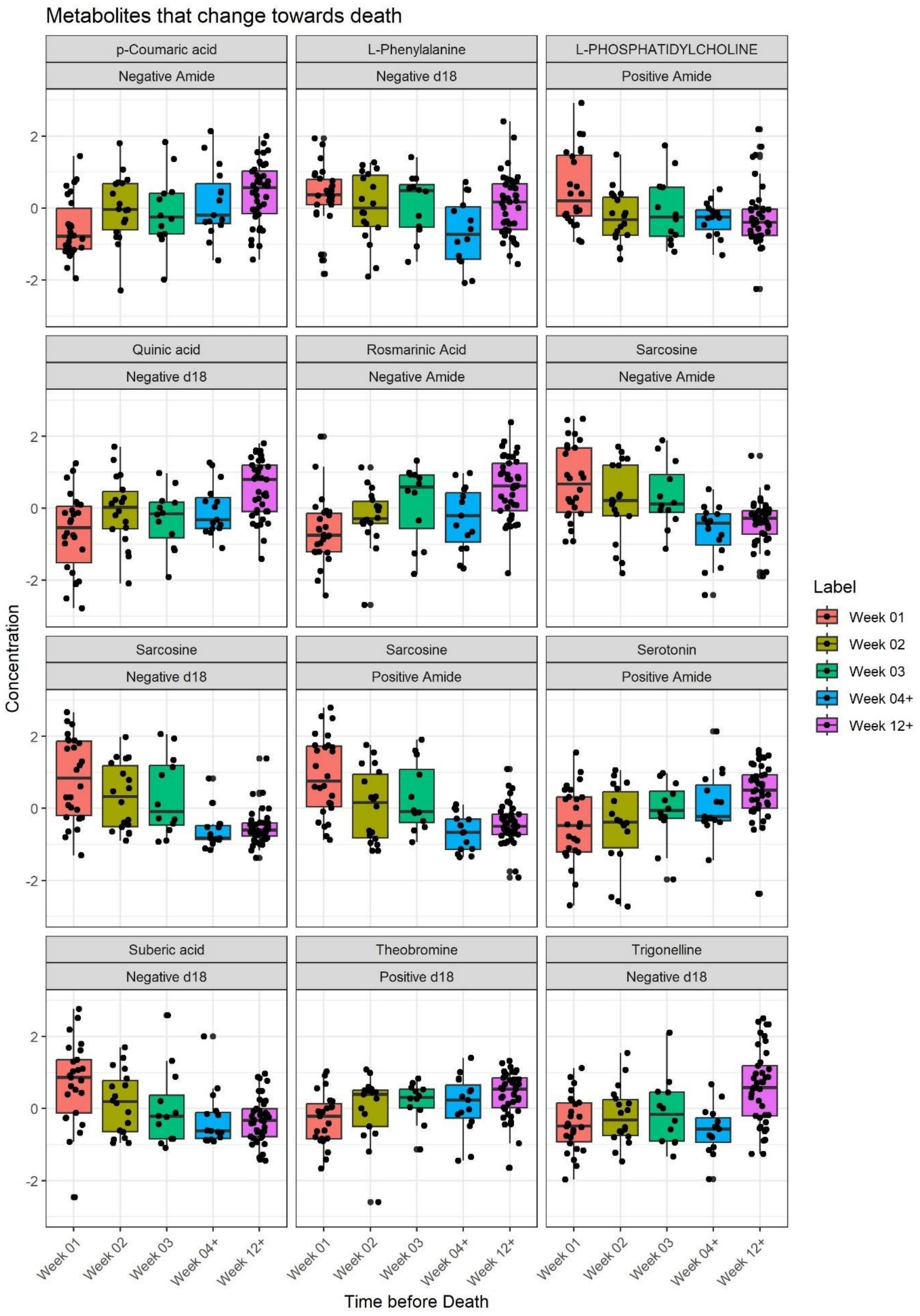

Supplementary Figure 8

Comparison of each metabolite identified by ANOVA over the last 12 weeks of life versus more than 3 months from death.

Plots for each metabolite identified as significant by ANOVA (i.e. FDR adjusted p-value <0·05.). Metabolites were identified from different LC-QTOF-MS protocols (A = Amide column, D= DC18 column, '+' = Positive mode, '-' = Negative mode). Each metabolite is compared over the last 12 weeks of life versus more than 3 months from death. Week 01 (n=26), 02 (n=18) and 03 (n=12) on the x-axis indicates patients' measurement in the last week, 2 weeks and 3 weeks of life; Week 04+ (n=15) indicates measurements from week 4 to week 11; Week 12+ (n=41) indicates more than 3 months before death. Data were normalized by reference feature, log-transformed and auto scaled (centered around the mean and divided by the standard deviation of each variable).

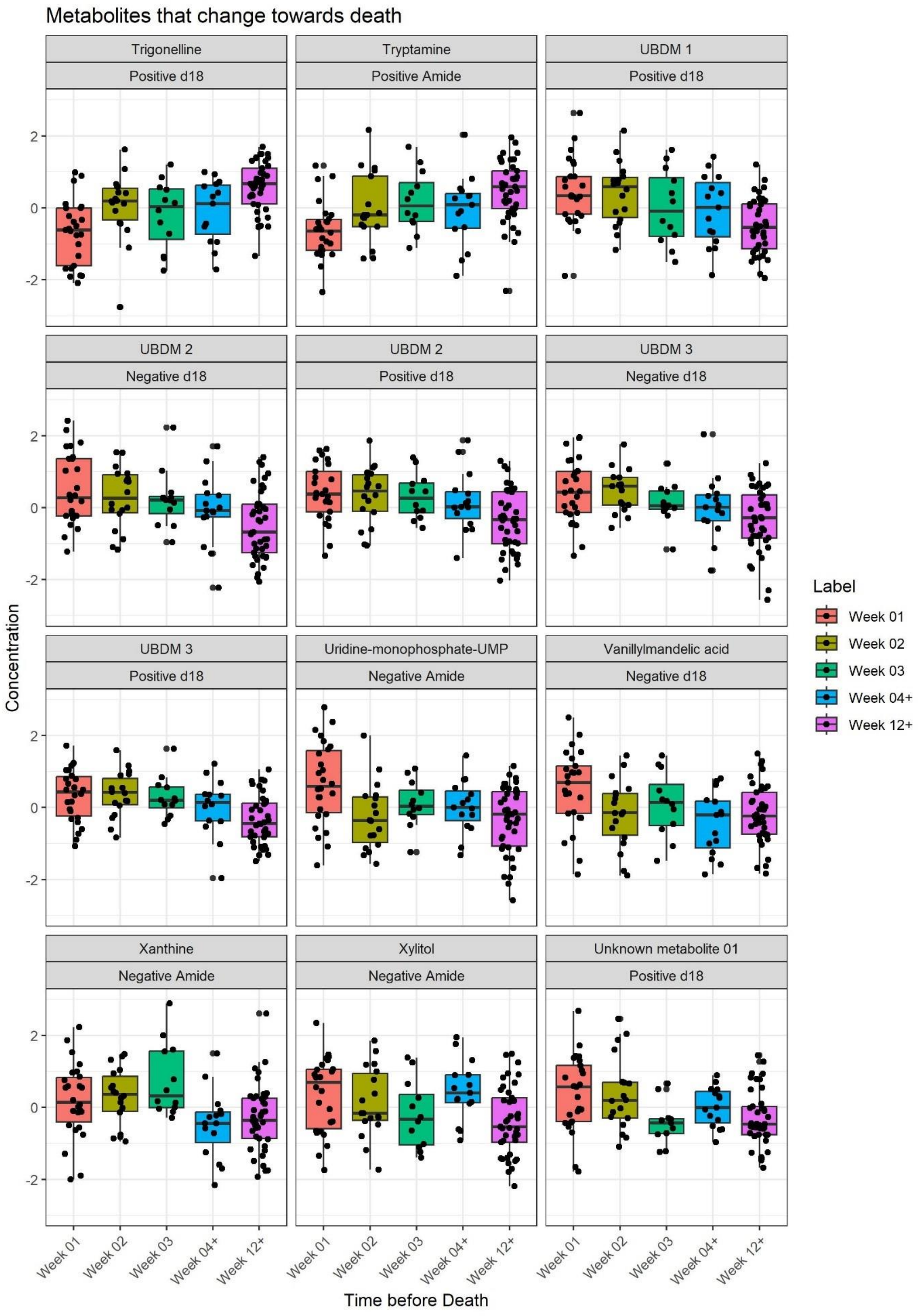

Supplementary Figure 9

Comparison of each metabolite identified by ANOVA over the last 12 weeks of life versus more than 3 months from death.

Plots for each metabolite identified as significant by ANOVA (i.e. FDR adjusted p-value <0·05.). Metabolites were identified from different LC-QTOF-MS protocols (A = Amide column, D= DC18 column, '+' = Positive mode, '-' = Negative mode). Each metabolite is compared over the last 12 weeks of life versus more than 3 months from death. Week 01 (n=26), 02 (n=18) and 03 (n=12) on the x-axis indicates patients' measurement in the last week, 2 weeks and 3 weeks of life; Week 04+ (n=15) indicates measurements from week 4 to week 11; Week 12+ (n=41) indicates more than 3 months before death. Data were normalized by reference feature, log-transformed and auto scaled (centered around the mean and divided by the standard deviation of each variable).

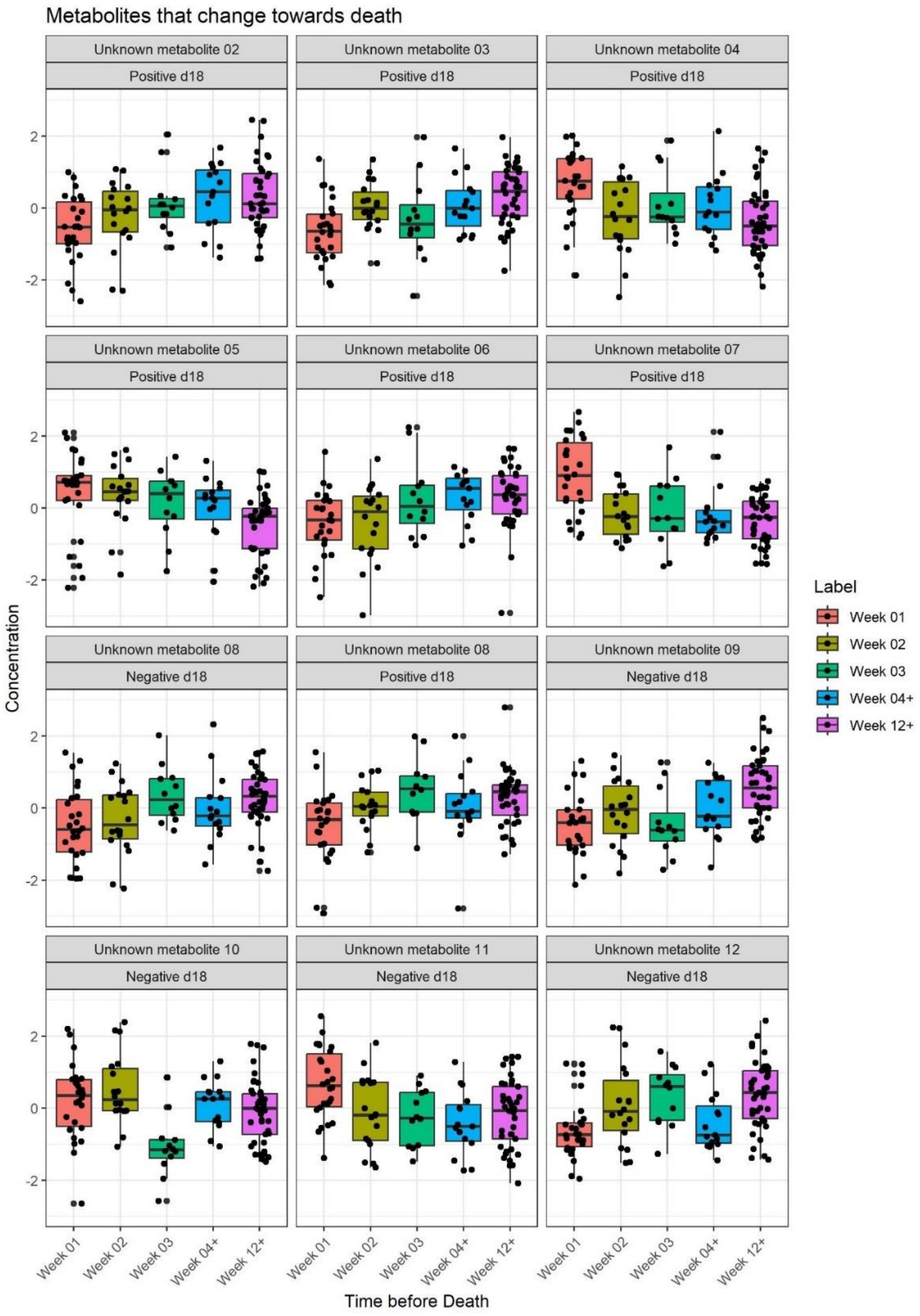

Supplementary Figure 10

Comparison of each metabolite identified by ANOVA over the last 12 weeks of life versus more than 3 months from death.

Plots for each metabolite identified as significant by ANOVA (i.e. FDR adjusted p-value <0·05.). Metabolites were identified from different LC-QTOF-MS protocols (A = Amide column, D= DC18 column, ‘+’ = Positive mode, ‘-’ = Negative mode). Each metabolite is compared over the last 12 weeks of life versus more than 3 months from death. Week 01 (n=26), 02 (n=18) and 03 (n=12) on the x-axis indicates patients’ measurement in the last week, 2 weeks and 3 weeks of life; Week 04+ (n=15) indicates measurements from week 4 to week 11; Week 12+ (n=41) indicates more than 3 months before death. Data were normalized by reference feature, log-transformed and auto scaled (centered around the mean and divided by the standard deviation of each variable).

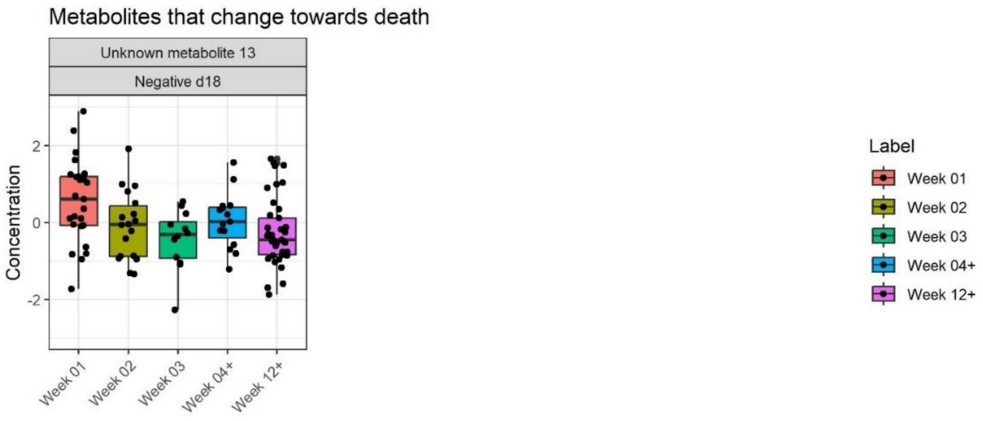

Time before Death

Supplementary Figure 11

Calibration curves for the 30 day Cox lasso regression model at days 10, 20 and 30 in the Training cohort and Validation cohort.

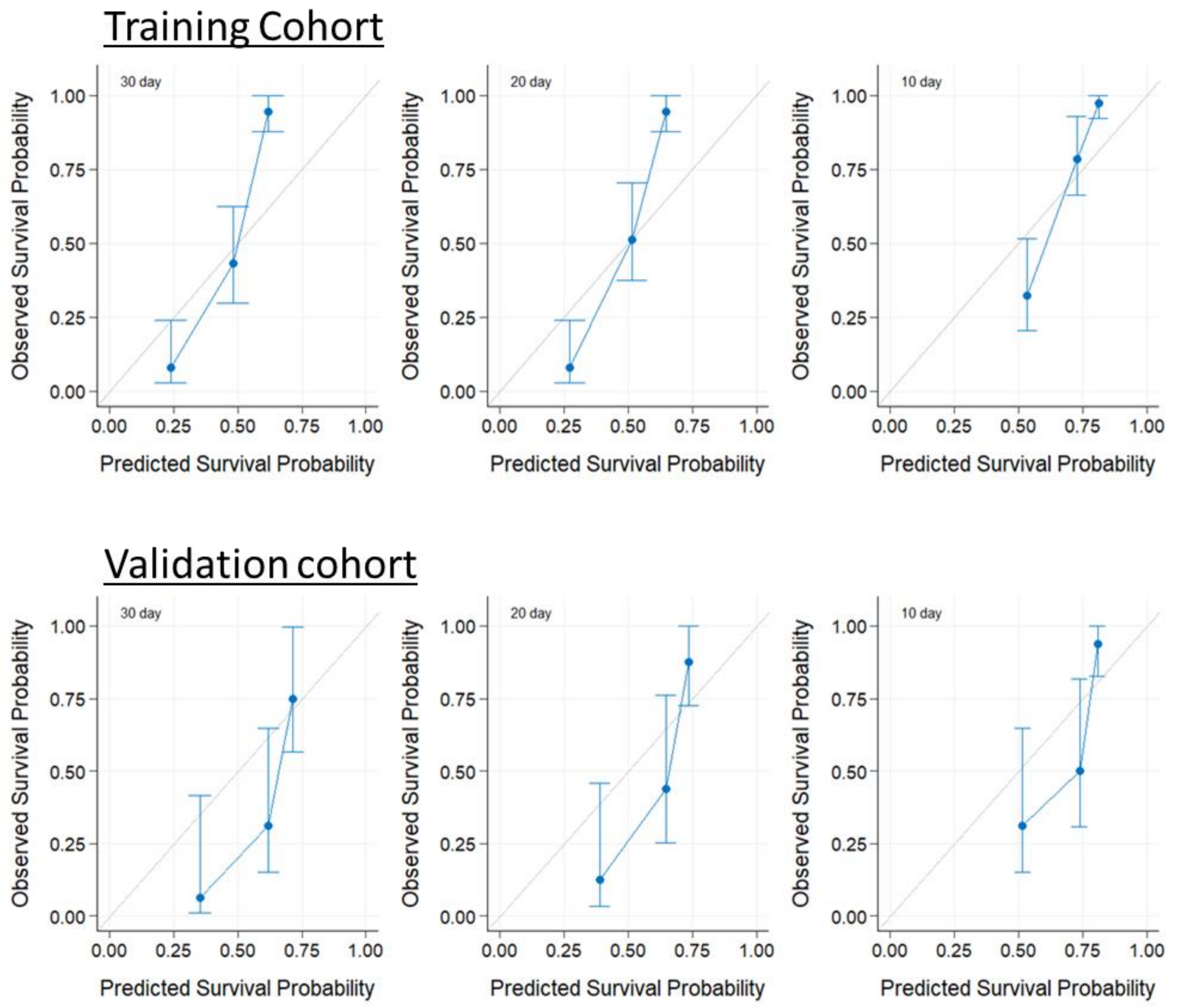

Supplementary Table 1

Table of molecular mass and Retention Time (RT) for the Unknown metabolites.

**Metabolite name**	**Mass**	**RT**
Unknown metabolite 1	194·0426	1·3
Unknown metabolite 2	188·079	1·74
Unknown metabolite 3	129·0801	1·72
Unknown metabolite 4	295·142	4·31
Unknown metabolite 5	110.0368	4.65
Unknown metabolite 6	304·0794	4·28
Unknown metabolite 7	274·1148	3·48
Unknown metabolite 8	225·0638	4·77
Unknown metabolite 9	179·0787	1·22
Unknown metabolite 10	194·0425	1·29
Unknown metabolite 11	117·0793	1·64
Unknown metabolite 12	212·068	5·22
Unknown metabolite 13	122·0368	5·95
Unknown metabolite 14	205·0738	6·77
Unknown metabolite 15	195·0538	4·43

Supplementary Table 2

MS2 Spectra data for Unknown metabolite 5 (neutral mass 188.0472, RT 6.77, ESI+)

Collision energy 10

m/z	Abund	Abund %
157.086	1639.91	100
83.0856	377.38	23.01
139.0752	231.65	14.13
111.0802	192.27	11.72
129.0908	175.01	10.67
192.1037	91.3	5.57
191.1066	90.7	5.53
191.0701	86.32	5.26
120.0808	63.35	3.86
128.1059	52.53	3.2

Collision energy 20

m/z	Abund	Abund %
83.0856	825.84	100
55.054	296.4	35.89
91.0542	141.02	17.08
111.0799	85.95	10.41
131.0851	80.47	9.74
93.0702	64.52	7.81
157.0851	62.39	7.55
120.0802	60.58	7.34
133.0282	47.05	5.7
117.0695	46.06	5.58

Collision energy 40

m/z	Abund	Abund %
55.0538	270.18	100
41.0384	142.29	52.66
115.0538	97.31	36.01
55.0178	77.16	28.56
65.0383	61.19	22.65
45.0331	60.02	22.21
89.0388	59.34	21.96
69.0697	57.85	21.41
105.0691	48.84	18.08
43.0175	47.37	17.53

Supplementary Table 3

MS2 Spectra data for Unknown metabolite 7 (neutral mass 274.1148, RT 3.48, ESI+)

Collision energy 10

m/z	Abund	Abund %
45.0445	319.34	100
275.1661	306.15	95.87
206.049	253.91	79.51
242.0119	210.48	65.91
200.9845	204.83	64.14
277.1194	104.27	32.65
275.1041	102.3	32.03
257.1118	100.48	31.46
277.0841	96.11	30.1
160.0424	92.98	29.12

Collision energy 20

m/z	Abund	Abund %
85.0286	625.19	100
130.05	402.81	64.43
114.0548	389.12	62.24
121.028	340.07	54.39
115.0381	281.62	45.05
200.9849	173.05	27.68
114.0635	154.87	24.77
276.148	153.71	24.59
84.0445	153.36	24.53
122.0283	143.63	22.97

Collision energy 40

m/z	Abund	Abund %
84.0442	256.68	100
43.0178	253.57	98.79
93.0334	250.08	97.43
68.0493	206.83	80.58
45.0445	202.21	78.78
121.0283	201.38	78.46
55.0179	166.79	64.98
60.0803	112.93	44
86.0597	106.35	41.43
85.0643	98.24	38.27

Supplementary Table 4

Metabolites identified for pathway analysis

LC-QTOF-MS Technique	Metabolites identified using LC-QTOF-MS	Metabolites identified using KEGG	Metabolites used in the pathways	
			2 Weeks	3 Days
DC18 Positive mode	209	154	102	148
DC18 Negative mode	182	150	108	124
Amide Positive mode	126	114	60	63
Amide Negative mode	111	97	35	55

The total number of metabolites identified, the number identified by KEGG and the number of metabolites used in the pathway analysis for the last 2 weeks and last 3 days from the different LC-QTOF-MS analysis approaches. The number of metabolites is the number of metabolites that match the pathways rather than the absolute number of metabolites that match to the pathways. Several metabolites will be present in more than one pathway.

Supplementary Table 5

KEGG Pathway analysis

**a)** **2 Weeks from death**	**b)** **2 Weeks / 3 days**			**c)** **3 Days from death**
Disturbed‡<br>– Propanoate metabolism<br>– Pyruvate metabolism<br>– Valine, leucine and isoleucine biosynthesis<br>– Vitamin B6 metabolism†	Increased<br>– β-Alanine metabolism<br>– Alanine, aspartate and glutamate metabolism†<br>– Arginine biosynthesis<br>– Arginine and proline metabolism<br>– Cysteine and methionine metabolism†<br>– Folate biosynthesis<br>– Galactose metabolism<br>– Glycine, serine and threonine metabolism<br>– Histidine metabolism†<br>– Lysine degradation<br>– Nicotinate and nicotinamide metabolism<br>– One carbon pool by folate<br>– Pantothenate and CoA biosynthesis†<br>– Phenylalanine, tyrosine and tryptophan biosynthesis<br>– Purine metabolism†<br>– Pyrimidine metabolism†<br>– Sphingolipid metabolism<br>– Starch and sucrose metabolism<br>– Synthesis and degradation of ketone bodies<br>– Taurine and hypotaurine metabolism†<br>– Tryptophan metabolism†<br>– Vitamin B6 metabolism†	Decreased<br>– Ascorbate and aldarate metabolism†<br>– Fructose and mannose metabolism<br>– Glycerolipid metabolism<br>– Glycerophospholipid metabolism<br>– Glycosylphosphatidylinositol (GPI)-anchor biosynthesis<br>– Pentose and glucuronate interconversions†<br>– Pentose phosphate pathway<br>– Pyrimidine metabolism†<br>– Tyrosine metabolism†<br>– Valine, leucine and isoleucine degradation	Disturbed‡<br>– Alanine, aspartate and glutamate metabolism†<br>– Aminoacyl-tRNA biosynthesis<br>– Amino sugar and nucleotide sugar metabolism†<br>– Ascorbate and aldarate metabolism<br>– Butanoate metabolism<br>– Cysteine and methionine metabolism†<br>– Glycolysis / Gluconeogenesis<br>– D-Glutamine and D-glutamate metabolism<br>– Glyoxylate and dicarboxylate metabolism<br>– Histidine metabolism†<br>– Inositol phosphate metabolism<br>– Nitrogen metabolism<br>– Pantothenate and CoA biosynthesis†<br>– Pentose and glucuronate interconversions†<br>– Phenylalanine metabolism<br>– Purine metabolism†<br>– Tryptophan metabolism‡<br>– Tyrosine metabolism†	Increased<br>– Amino sugar and nucleotide sugar metabolism†<br>– Citrate cycle (TCA cycle)<br>– Galactose metabolism†<br>– Phosphonate and phosphinate metabolism<br>– Steroid hormone biosynthesis<br><br>Disturbed‡<br>– Arginine biosynthesis<br>– Biotin metabolism<br>– Galactose metabolism†<br>– Primary bile acid biosynthesis<br>– Taurine and hypotaurine metabolism†

Biochemical pathways that change significantly in a) the last 2 weeks, b) shared in the last 2 weeks and last 3 days and c) the last 3 days of life. Different pathways were identified from different LC-QTOF-MS protocols (A Amide column, D DC18 column, + Positive mode, - Negative mode). † denotes a duplicated pathway identified using different methods. ‡Disturbed represents a pathway where some metabolites increased and some metabolites decreased in abundance. P was <0·05.
