# Supplementary material for: Urinary metabolite model to predict the dying process in lung cancer patients
Source: Commun Med (Lond). 2025 Feb 27;5:49. doi: 10.1038/s43856-025-00764-3 (PMC11868640; doi:10.1038/s43856-025-00764-3)
Supplement: Supplementary file 3 — Description of Additional Supplementary Files [file 43856_2025_764_MOESM3_ESM.pdf]

Description of Additional Supplementary Files

File name: Supplementary DATA 1

File description: Table of metabolites that show an increase (↑) or decrease (↓) in abundance towards death identified from ANOVA analysis as significant in the last weeks of life

File name: Supplementary DATA 2

File description: Summary table of metabolites that INCREASE over 2-fold at different time intervals in the last month of life.

File name: Supplementary DATA 3

File description: Summary table of metabolites that DECREASE over 2-fold at different time intervals in the last month of life.

File name: Supplementary DATA 4

File description: This table is a summary of all the metabolites identified as changed significantly from the volcano plot analysis and ANOVA analysis of the Training dataset. It includes a summary of the KEGG Pathway analysis for the last 2 weeks and last 3 days. For each metabolite, a greater than 2-fold change in last 4 weeks, 2 weeks, 5 days and last 3 days of life identified by volcano plot analysis is presented in parenthesis. * The change is shown in the ANOVA graphs, Supplementary Figure 1. ‡ Disturbed represents a pathway where some metabolites increased and some metabolites decreased in abundance.

File name: Supplementary DATA 5

File description: Source data is located in Supplementary Data 5.
